# Control of fire blight (*Erwinia amylovora*) on apple trees with trunk-injected plant resistance inducers and antibiotics and assessment of induction of pathogenesis-related protein genes

**DOI:** 10.3389/fpls.2015.00016

**Published:** 2015-02-10

**Authors:** Srđan G. Aćimović, Quan Zeng, Gayle C. McGhee, George W. Sundin, John C. Wise

**Affiliations:** ^1^Tree Fruit Pathology Laboratory, Department of Plant, Soil and Microbial Sciences, Michigan State UniversityEast Lansing, MI, USA; ^2^Applied Insecticide Toxicology Laboratory, Department of Entomology, Michigan State UniversityEast Lansing, MI, USA

**Keywords:** trunk injection, fire blight, apple disease control, antibiotics, SAR induction, PR genes, acibenzolar-*S*-methyl, potassium phosphites

## Abstract

Management of fire blight is complicated by limitations on use of antibiotics in agriculture, antibiotic resistance development, and limited efficacy of alternative control agents. Even though successful in control, preventive antibiotic sprays also affect non-target bacteria, aiding the selection for resistance which could ultimately be transferred to the pathogen *Erwinia amylovora*. Trunk injection is a target-precise pesticide delivery method that utilizes tree xylem to distribute injected compounds. Trunk injection could decrease antibiotic usage in the open environment and increase the effectiveness of compounds in fire blight control. In field experiments, after 1–2 apple tree injections of either streptomycin, potassium phosphites (PH), or acibenzolar-*S*-methyl (ASM), significant reduction of blossom and shoot blight symptoms was observed compared to water injected control trees. Overall disease suppression with streptomycin was lower than typically observed following spray applications to flowers. Trunk injection of oxytetracycline resulted in excellent control of shoot blight severity, suggesting that injection is a superior delivery method for this antibiotic. Injection of both ASM and PH resulted in the significant induction of PR-1, PR-2, and PR-8 protein genes in apple leaves indicating induction of systemic acquired resistance (SAR) under field conditions. The time separating SAR induction and fire blight symptom suppression indicated that various defensive compounds within the SAR response were synthesized and accumulated in the canopy. ASM and PH suppressed fire blight even after cessation of induced gene expression. With the development of injectable formulations and optimization of doses and injection schedules, the injection of protective compounds could serve as an effective option for fire blight control.

## INTRODUCTION

*Erwinia amylovora* (Burrill 1882) Winslow et al. (1920) is a devastating bacterial pathogen of plant species in the *Rosaceae* family, causing the disease fire blight. Yearly losses due to fire blight can be substantial in many countries worldwide. In the USA, fire blight losses and control costs per year are estimated to be more than $100 million (Norelli et al., 2003). In Michigan, a fire blight epidemic in 2000 resulted in economic losses of $42 million due to removal of approximately 400,000 apple trees (Longstroth, 2001). In Washington and northern Oregon, economic losses on pome fruits due to fire blight were over $68 million (Stockwell et al., 2002).

Control of fire blight is difficult because currently there are no available synthetic compounds with systemic properties that directly affect the pathogen and that could improve fire blight protection programs. An added difficulty in fire blight management is the occurrence and spread of strains of *E. amylovora* with resistance to the antibiotic streptomycin, limiting the efficacy of this compound as a plant disease control agent (Chiou and Jones, 1993; McGhee and Sundin, 2011). Research shows that when non-target bacterial populations are exposed to broadcast applications of streptomycin in the agroecosystem, resistance genes can be selected, and can then be transferred to target pathogens, such as *E. amylovora* (Chiou and Jones, 1993; Sundin et al., 1995). The use of antibiotics for plant protection is under scrutiny due to concerns that spray-drifted antibiotics impact the environment and soil, with commensal microorganisms as reservoirs of genes for antibiotic resistance important in clinical medicine (McManus, 2014). Even though so far described mechanisms for transfer of genes for antibiotic resistance are distinct in plant and human pathogens (McManus et al., 2002; Sundin, 2002), the fear of potential transfer of antibiotic resistance from plant to human pathogenic bacteria or other bacteria in nature drives the scrutiny on antibiotic use for plant protection and aims to preserve their efficiency in human medicine (Sundin et al., 1995; Sundin and Bender, 1996; McManus et al., 2002; McManus, 2014).

Traditionally, management of fire blight relies on cultural practices and preventive copper and antibiotic sprays (Norelli et al., 2003). Preventive timing of sprays provides direct contact of applied materials with the pathogen, either before or immediately after the pathogen reaches the apple flowers or shoots. Therefore, successful fire blight management is dependent on controlling the epiphytic pathogen populations. Once *E. amylovora* enters the host xylem or cortical parenchyma and spreads in the endophytic phase of pathogenesis (Koczan et al., 2009), external control methods become ineffective. In the past two decades, scientific research addressing issues in fire blight management has focused on investigating plant resistance inducers and biological control agents as potential alternatives to antibiotics and copper (Johnson and Stockwell, 1998; Maxson-Stein et al., 2002; Norelli et al., 2003; Sundin et al., 2009; Johnson and Temple, 2013). However, both strategies showed limited efficiency in fire blight control in some environments (Thomson et al., 1998) and frequent reapplication is often required, making them more costly and labor intensive. As a result, plant resistance inducers, for example, are currently viewed solely as a supplement to antibiotic programs.

In regards to protection of orchards, air blast ground sprayers are inefficient in topical compound delivery with pesticide solution losses into the environment of up to 44–71% (Steiner, 1969). Furthermore, even under the best spray coverage, activity of topically applied protective products is negatively affected by variable weather conditions (rainfall, sunlight, temperature), specific properties of the phyllosphere, and a limited rate of absorption and subsequent movement in the plant (Windels and Lindow, 1985; Percival, 2001; Gozzo, 2003; Christiano et al., 2010; Cabrefiga et al., 2011). These difficulties bring into question the means by which materials are delivered for fire blight control and support investigations into alternative solutions (McManus et al., 2002; Düker and Kubiak, 2011).

Trunk injection is an alternative approach for delivery of plant protective compounds in tree fruit crops. It harnesses the vascular transport capacity of a tree, which allows active ingredient (a.i.) translocation and subsequent distribution into the canopy where protection is needed (Percival and Boyle, 2005; Aćimović et al., 2014b). The majority of tree injection technologies are based on compound delivery into the xylem. Trunk injection was originally developed and is widely used for the purposes of efficient plant protection and nutrition in landscape tree care, offering numerous advantages that could enhance disease management of fruit trees (Byrne et al., 2014; VanWoerkom et al., 2014; Wise et al., 2014). Most importantly, trunk injection is a precise pesticide delivery system, facilitating compound deployment without direct pesticide losses into the environment. These properties could be particularly effective for control of *E. amylovora*, which spreads through the xylem and cortical parenchyma (Bogs et al., 1998; Perino et al., 1999). In landscapes, trunk injection of bactericides and biopesticides such as oxytetracycline and potassium phosphites is used for fire blight control on sensitive varieties of crabapples (*Malus* spp.) and ornamental pears (*Pyrus* spp.; www.arborjet.com). We found that trunk-injected oxytetracycline in apples provides control of fire blight incidence of 60%, well surpassing kasugamycin and copper chelate effects (Aćimović et al., 2014a). In the current study we investigated the effect of oxytetracycline on shoot blight severity in apples.

However, research on trunk injection of bactericides and plant resistance inducers demonstrating effectiveness in disease control on apples is very limited. Previous investigations have shown that trunk injection of potassium phosphites, i.e., mono- and di-potassium salts of phosphorous acid (Arborfos^TM^, Mauget Inc., Arcadia, CA, USA) on ‘Paula Red’ apple trees provided significant reduction of shoot blight, while the injected prohexadione-calcium (Apogee^®^, BASF Corp., Research Triangle Park, NC, USA) was ineffective (Spitko, 2008; Aćimović et al., 2014a). In a separate study on ‘Gala Must’ and ‘White Transparent’ apples, trunk-injected prohexadione-carboxylic acid (PCA) provided significant control of *E. amylovora* infections on flowers, comparable to the sprayed streptomycin (Düker and Kubiak, 2011). However, none of these or other previous studies demonstrated whether sprayed or injected potassium phosphites or injected plant resistance inducers activate the synthesis of defensive plant pathogenesis related (PR) proteins, which are defined as plant host proteins produced only in response to attack by pathogens or by a related event (van Loon et al., 1994). This is one of the primary modes of action of systemic acquired resistance (SAR) inducers in plant disease suppression. The SAR is a form of induced resistance in plants with a specific defense signaling pathway. SAR occurs after localized exposure to a pathogen or alternatively, after spraying with a synthetic or natural compound, commonly known as an inducer (Hammerschmidt, 2007). Demonstrating the expression of PR genes such as PR-1, PR-2, PR-5, PR-8, PR-10, and others, after application of a resistance inducer has been widely accepted as a hallmark of plant defensive SAR induction (Brisset et al., 2000; Ziadi et al., 2001; Bonasera et al., 2006; Ebrahim et al., 2011). The most commonly screened PR genes expressed in apples and other plant-pathogen systems are PR-1 (anti-oomycete activity), PR-2 (β-1,3-glucanase), and PR-8 (class III chitinase; Maxson-Stein et al., 2002). These genes were expressed in leaves of apple seedlings after sprays of acibenzolar-*S*-methyl (ASM; Maxson-Stein et al., 2002). However, on 1 year-old apple trees, only inoculation with *E. amylovora* but not the sprayed ASM induced PR gene expression in shoots (Bonasera et al., 2006). In the present study, we uniquely investigated the effect of trunk-injected and not sprayed plant resistance inducer, potassium phosphites, and bactericides on the development of fire blight and for the first time we correlated disease suppression and PR gene expression on mature apple trees. Demonstrating whether potassium phosphites induce PR gene expression was of particular interest since previous studies lack this evidence, even though claiming plant resistance induction (Gozzo and Faoro, 2013).

The hypothesis of our study was that significant control of fire blight and expression of PR genes could be achieved by 1–2 trunk injections of maximum seasonally allowed or lower doses of antibiotics, potassium phosphites, and plant resistance inducers. Our objective was twofold: (1) Assess the performance of trunk-injected antibiotics, potassium phosphites, and plant resistance inducers in control of *E. amylovora* on apple blossoms and shoots, and (2) Determine whether trunk-injected plant resistance inducers and potassium phosphites were capable of inducing the expression of PR genes in apple leaves and flowers, as markers of SAR response. Our goal was to evaluate whether trunk injection could enhance the activity of protective compounds in fire blight control, and specifically the activity of plant resistance inducers.

## MATERIALS AND METHODS

### TRUNK INJECTIONS

Four cardinally oriented injection ports per tree, positioned approximately 10–15 cm above the ground level, were created by drilling 25.4 mm into the xylem tissue and 9.53 mm in diameter, with a cordless 1500 rpm drill (DeWalt Industrial Tool Co., Baltimore, MD, USA; Aćimović et al., 2014b). Ports were sealed with Arborplug^®^ no. 4 (Arborjet Inc., Woburn, MA, USA), using screwdriver-like plug tapper and a hammer, with plug positioned just below the bark level to allow port closure with cambium.

#### Blossom and shoot blight incidence control injections

Due to cold weather conditions, a poorly developed leaf canopy and resulting weak transpiration pull of sap in the wood xylem, injections on March 26, 2012 were conducted with Viper air/hydraulic micro-injection system^®^ (Arborjet Inc., Woburn, MA). The Viper system allowed quick injection of large solution volumes per tree under 110 psi of air pressure thus compensating for weak transpiration pull in xylem due to underdeveloped tree canopy. Because of warm weather conditions and good transpiration facilitated by well-developed leaf canopy which increases the sap pull in the xylem, trunk injections on April 23, 2012, May 1, 2013, and May 22, 2013 were conducted with Tree IV^®^ air/hydraulic micro-injection system (Arborjet Inc., Woburn, MA). The Tree IV system facilitated fast injection of large solution volumes under fixed 60 psi of air pressure. Injection needles of these devices were inserted through the septum in the Arborplugs^®^ thus allowing delivery of protective solutions through the ports. Total injected volume per tree was divided equally among the four ports.

#### Shoot blight severity control injections

Trunk injections were conducted in a similar manner described above by using a Quik-jet^®^ micro-injection system (Arborjet Inc., Woburn, MA, USA). We used the Quik-jet system which relies solely on hand-generated hydraulic pressure to quickly inject low solution volumes into each port. Injection ports were created and sealed using the same method described above.

### CONTROL OF BLOSSOM AND SHOOT BLIGHT

#### Chemical materials used in blossom and shoot blight incidence control

Orchard experiments were conducted in 2012 and 2013 at one of two Michigan State University (MSU) research stations. To prove that hypothesized effects occur on mature apple trees we independently replicated this experiment on ‘Gala’ trees of two different ages. In 2012, research was conducted at the MSU Plant Pathology Farm in East Lansing, Michigan, using 14 year-old ‘Gala’ apple trees, *Malus domestica* Borkh. Trees were trunk-injected with compounds using dosages listed in **Table 1**. Injections in 2012 were conducted on March 26, at the tight cluster stage in apples or 21 days before 80% bloom, and on April 23, at petal fall. In 2013, experiments were conducted at the MSU Trevor Nichols Research Center in Fennville, Michigan, using 21 year-old ‘Gala’ apple trees. Injections were conducted on May 1, at transition of half inch green to tight cluster (or 13 days before 80% bloom), and on May 22, at petal fall using the same doses in **Table 1**. In both years, treatment ASM 1 was injected only on the first date (**Table 1**). Except for potassium salts of phosphorous acid (PH, potassium phosphites) each dose of a product was injected with 520 ml of water per tree for dilution and to ease translocation in xylem. Used doses per tree were equivalent to the US EPA label rates for either maximum amount allowed per one season per 0.405 ha with 250 apple trees, one half of that rate, one spray treatment per 0.405 ha with 250 apple trees (**Table 1**), or determined based on previous trunk injection research with generic products (Spitko, 2008). Water injected trees served as a control, and non-injected non-inoculated trees were also used as controls.

**Table 1:** Trunk-injected compounds on apple trees for control of blossom and shoot blight in 2012 and 2013.

Treatment	Active ingredient (a.i.)	Dose
**Blossom and shoot blight incidence control on ‘Gala’ apple trees**
ASM 1	Acibenzolar-*S*-methyl 50% (Actigard, Syngenta, AG)	1 × 0.34 g/tree
ASM 2		2 × 0.34 g/tree
PH	Mono- and di-potassium salts of phosphorous acid 45.8% (Phosphojet, Arborjet, Inc.)	2 × 22.5 ml/tree
SS	Streptomycin sulfate 22.4%/17% streptomycin/(Agrimycin, Nufarm, Ltd.)	2 × 1.82 g/tree
Water injected control	–	2 × 520 ml/tree
**Shoot blight severity control on ‘Jonathan’ apple trees**
OTC	Oxytetracycline hydrochloride (ArborBiotic^TM^, MFG Scientific Inc.)	1 × 0.28 g + 2.52 ml of water/each 25.4 mm of DFH*
Water injected control	–	2.52 ml of water/each 25.4 mm of DFH

*DFH, trunk diameter at 30.5 cm height.

In 2012, four replicate trees per treatment were arranged in a randomized complete block design. Blocking compensated for variable crown sizes in trees (large, medium, medium–small, and small) since different transpiring leaf areas could modulate the speed of compound translocation and accumulation in the canopy after injection (Harrell, 2006). In 2013, four replicate trees per treatment were arranged in a completely randomized design (CRD) because the trees were very uniform in crown size.

#### Inoculation of flowers and disease evaluation

Late in the afternoon on April 16, 2012 (80% bloom), apple flowers were spray-inoculated with a suspension of *E. amylovora* strain Ea110 (5.4 × 10^6^ CFU/ml) in distilled water using a hand-sprayer (Solo 457, 11.36 L, Solo Inc. Newport News, VA, USA). Between the hours of 18 and 24 on May 14, 2013 (80% bloom), flowers were spray-inoculated with Ea110 at 0.7 × 10^6^ CFU/ml. We used different inoculum densities in 2012 and 2013 because these 2 years differed significantly in favorability of weather conditions for inoculation, fire blight establishment, and development.

Blossom blight incidence was evaluated on May 22, 29 and June 5, 2012, and on June 11, 18, and 25, 2013. We randomly chose blossom clusters on spurs and counted the number of diseased and healthy blossom clusters in a 100-cluster sample. Blossom blight incidence was calculated as blossom blight percent on a per tree basis. Because *E. amylovora* migrated from infected flowers into the intensively growing shoots, blossom blight driven shoot blight incidence was evaluated on May 29 and June 5, 2012. Shoot blight was rated only two times in 2012 since there was no change in disease incidence before or after these two rating dates. In 2013, shoot blight incidence was evaluated at the same time when blossom blight was rated; June 11, 18, and 25. After counting a 100-shoot per tree random sample the shoot blight incidence was calculated by comparing numbers of blighted and healthy shoots for each tree. For each treatment, blossom and shoot blight incidence means were calculated from four replicate trees.

#### Chemical materials used in shoot blight severity control

To prove that an injected compound can also affect severity of fire blight, orchard experiments were conducted in 2012 and 2013 at the Plant Pathology Research Farm in East Lansing, MI, USA. On April 23, 2012 (petal fall), mature 12 year-old ‘Jonathan’ apple trees were trunk-injected with oxytetracycline hydrochloride (OTC) using dose listed in **Table 1** and recommended by the US EPA label in landscape tree care (10% solution). Total dose per tree depended on each tree’s unique trunk diameter at one foot, i.e., 30.5 cm height (DFH). In 2013, apple trees previously injected in 2012 were re-injected on May 22, 2013 (petal fall) using the same dose in **Table 1**. Four replicate trees per treatment were arranged in a CRD.

#### Inoculation of shoots and disease evaluation

Shoot inoculations were conducted on May 7, 2012 and on May 30, 2013. The upper third of the leaf blade of second or the third youngest leaf on the shoot tip were removed with scissors dipped in *E. amylovora* (2012: 4.7 × 10^7^ CFU/ml; 2013: 5 × 10^8^ CFU/ml) suspended in 0.5X phosphate buffered saline (68.5 mM NaCl, 1.35 mM KCl, 5 mM Na_2_HPO_4_, 1 mM KH_2_PO_4_; Koczan et al., 2009). A total of 10 randomly chosen shoots per each ‘Jonathan’ tree were inoculated with *E. amylovora* strain Ea110, while additional 10 shoots on the same tree replicate were inoculated with distilled water as a negative control. For each inoculated shoot, severity was calculated from the ratio of necrotic shoot length and total shoot length (cm). Total shoot length (cm) was recorded for negative control shoots. Measurements of total shoot length were first taken on May 6, 2012 and May 30, 2013, prior to inoculation. Shoot and necrosis lengths were then measured in 7 day intervals on May 14, 21, and 28 and on June 4, 11, and 18, 2012. 2013 measurements were recorded on June 10, 17, and 24 and on July 1, 8, and 15, 2013. Measurements ceased being collected when the terminal bud set on shoots. Shoot blight severity mean per tree (%) was calculated from 10 shoot replicates. Average shoot blight severity of each treatment was calculated from four replicate tree means.

### PR GENE EXPRESSION IN LEAVES AND FLOWERS

From injected ‘Gala’ apple trees for blossom blight control in 2012 and 2013, 21 leaves and 21 flowers per tree were collected for PR gene expression analysis. In 2012, leaves were collected on April 5 and 16, May 7 and 21, June 4 and 18, and July 2, and flowers were collected on April 16. In 2013, leaf samples were collected on May 10, 14, 23, and 31, and flowers on May 14. Samples were transported to the laboratory in a cooler with ice packs, then frozen in liquid nitrogen and stored at -80°C until RNA extraction. Out of four tree replicates per treatment in both years, samples from the same three were used for gene expression analysis through all sampling times. When the gene expression was found significant, in both years we chose the most representative treatment replicates to show the relative gene expression in results section.

In both years, gene expression analyses were conducted for water injected control, non-injected non-inoculated control and ASM and PH treatments (**Table 1**). One hundred milligram of vegetative tissue was ground in liquid nitrogen for each leaf and flower sample. RNA was extracted from tissue using an E.Z.N.A. Plant RNA kit (Omega Bio-Tek Inc., Norcross, GA, USA; Plant RNA Protocol II for difficult samples) following manufacturer’s instructions. RNA purification was conducted with a TURBO DNA-free kit (Ambion, Life Technologies Corp., Carlsbad, CA, USA). All RNA samples were diluted to the lowest sample RNA concentration. cDNA was synthesized with TaqMan Reverse Transcription kit (Invitrogen, Life Technologies Corp., Carlsbad, CA, USA) in PTC-100 Programmable Thermal Controller (MJ Research Inc., Waltham, MA, USA) using 25 μL reactions. RNA and DNA concentrations were determined with NanoDrop 1,000 Spectrophotometer (Thermo Fisher Scientific Inc., Wilmington, DE, USA). The expression levels of PR-1, PR-2, and PR-8 genes were quantified by qRT-PCR using a SYBR PCR Green Master Mix (Applied Biosystems Inc., Foster City, CA, USA) in a Step OnePlus Real-Time PCR machine (Applied Biosystems Inc., Foster City, CA, USA). The apple actin gene was used as an endogenous control (primers by Invitrogen, Life Technologies Corp., Carlsbad, CA, USA; Supplemental Table S1). For each biological replicate, i.e., apple tree, three technical replicates of PCR reactions were performed. Pair Wise Fixed Reallocation Randomization Test was used to compare the expression levels among the injection treatments (Multiple Condition Solver REST-MCS, version 2; Pfaﬄ, 2001).

### STATISTICAL ANALYSIS

Data were analyzed using MIXED procedure in SAS 9.3 (SAS Institute, 2012). Blossom blight data in 2012 were transformed using arcsine transformation to normalize the residuals. The main effects of treatment and time on blossom blight incidence in 2012 were analyzed using repeated measures best adjusted to spatial power variance covariance structure (α = 0.05). The main effects on shoot blight incidence in 2012 were analyzed using Type 3 Tests of Fixed Effects (*F* test, α = 0.05) for each time point independently. The main effects on blossom blight incidence in 2013 were analyzed with time as a fixed factor since no variance covariance structures reduced AIC and BIC criterions. The main effects on shoot blight incidence in 2013 were analyzed using repeated measures best adjusted using autoregressive covariance structure of first order.

Shoot blight severity data in 2012 were transformed using square root transformation to normalize the residuals. The main effects of OTC and time on shoot blight severity in 2012 were analyzed using repeated measures best adjusted to heterogeneous autoregressive variance covariance structure of first order (α = 0.05). In 2013, the main effects of OTC and time on shoot blight severity were analyzed using repeated measures best adjusted to spatial power variance covariance structure (α = 0.05). In all experiments tree was the subject of repeated measurements through time. When the main effects or their interaction were found to be statistically significant (*p* < 0.05), main effect and/or interaction slicing examination by main effects was performed, tested with *F* tests (α = 0.1 or 0.05), and pairwise or specific time or treatment comparisons were conducted using *t*-tests (α = 0.1 or 0.05) or Tukey’s HSD test (α = 0.05).

## RESULTS

### CONTROL OF BLOSSOM AND SHOOT BLIGHT INCIDENCE

**FIGURE 1:**
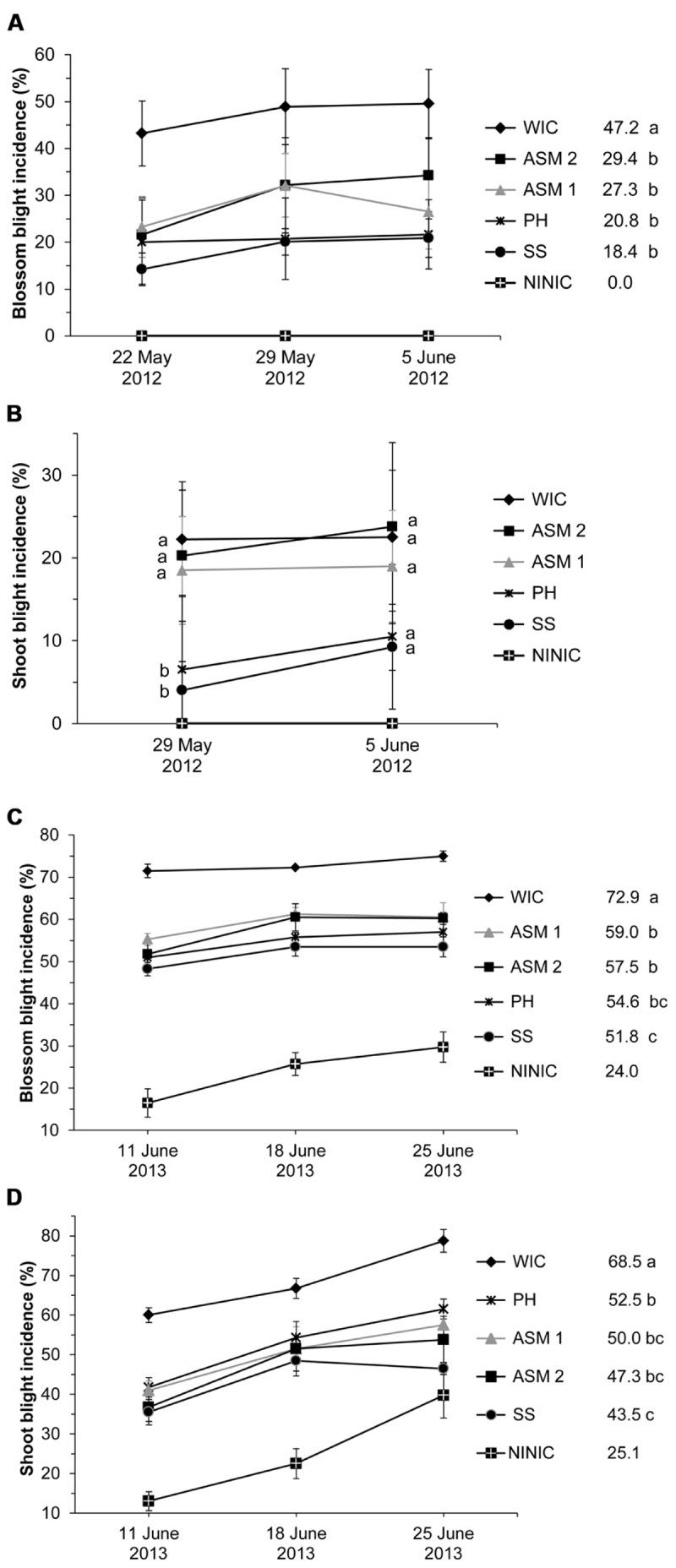
**Control of blossom and shoot blight incidence in 2012 (A,B) and in 2013 (C,D) after 1–2 trunk injections of ‘Gala’ apple trees with protective compounds**. WIC, water injected control; ASM, acibenzolar-*S*-methyl; PH, potassium salts of phosphorous acid (potassium phosphites); SS, streptomycin sulfate; NINIC, non-injected non-inoculated control. **(A,C)** Blossom blight incidence means across the three time points within one treatment followed by different letters are significantly different (*t*-test, 2012: *p* < 0.1, 2013: *p* < 0.05). **(B)** Shoot blight incidence means between treatments within one time point followed by different letters are significantly different (*t*-test, *p* < 0.1). **(D)** Shoot blight incidence means across the three time points within one treatment followed by different letters are significantly different (*t*-test, *p* < 0.05). Error bars represent SEM.

The injected compounds significantly affected blossom blight incidences in 2012 and 2013 (α = 0.1; α = 0.05) (Supplemental Table S2). At a medium infection pressure in 2012, injected streptomycin (SS) and PH reduced blossom blight and provided 61 and 55.9% disease control (*t*-test, α = 0.1; **Figure 1A**). ASM 1 and 2 provided statistically similar control to these compounds of 42.2 and 37.7%. At a high infection pressure in 2013, all injected compounds reduced blossom blight incidence but with different levels of control (**Figure 1C**). Injected SS provided blossom blight control of 28.9%, while PH followed with similar 25.1%. ASM 1 and 2 provided lower control of 19.1 and 21.1%, similar only to PH (**Figure 1C**).

Fire blight had spread from flowers onto the shoots in both 2012 and 2013 (**Figures 1B,D**). In 2012, the injected compounds affected shoot blight incidence only on 29 May (α = 0.1) when PH and SS provided good control of shoot blight incidence of 70.8 and 82%, while ASM 1 and 2 had no effect (*t*-test, α = 0.1; **Figure 1B**). Disease incidence did not change after June 5. In 2013, all injected compounds affected shoot blight incidence (α = 0.05; Supplemental Table S2; **Figure 1D**). SS was the most successful by providing shoot blight control of 36.5%. ASM provided weaker control of 27–30.9%, while PH gave only 23.4% (**Figure 1D**). PH was similar in effect to ASM 1 and 2. SS outperformed PH but was similar to ASM 1 and 2. In 2012, only PH caused limited phytotoxicity on 2 replicate trees with small and medium–small crown sizes, expressed as leaf narrowing and scorching of flowers and shoots on 1–3 branches per tree. This did not occur again in 2013.

Overall, the majority of injected compounds did not provide fire blight control levels on flowers and shoots between 92–99% usually achieved with spray application(s) of antibiotics in commercial apple orchards.

### CONTROL OF SHOOT BLIGHT SEVERITY

**FIGURE 2:**
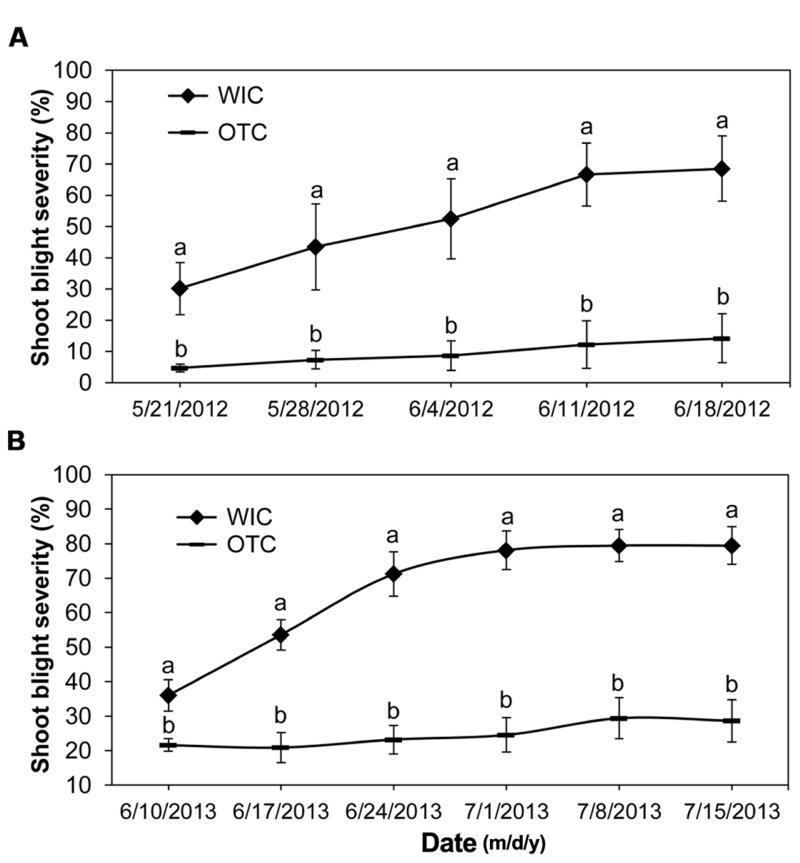
**Shoot blight control in 2012 (A) and 2013 (B) after single injection of ‘Jonathan’ apple trees with oxytetracycline per each year**. WIC, water injected control, OTC, oxytetracycline hydrochloride. Shoot blight severity means between treatments within one time point followed by different letters are significantly different (2012: Tukey’s HSD test, 2013: *t*-test, *p* < 0.05). Error bars represent SEM.

The main effect of injected OTC on shoot blight severity was significant for two years consecutively (α = 0.05; **Figure 2**). This antibiotic formulated for injection provided significant, season-long control of shoot blight after each injection (**Figure 2A**: interaction slicing examination by Tukey’s HSD test, and **Figure 2B**: interaction slicing examination by *t*-test, α = 0.05, Supplemental Table S2). In these experiments, fire blight necrosis significantly increased through time only in water injected control shoots (**Figure 2**). There were no signs of phytotoxicity observed after injection of OTC.

### PR GENE EXPRESSION

#### Gene expression in apple leaves

**FIGURE 3:**
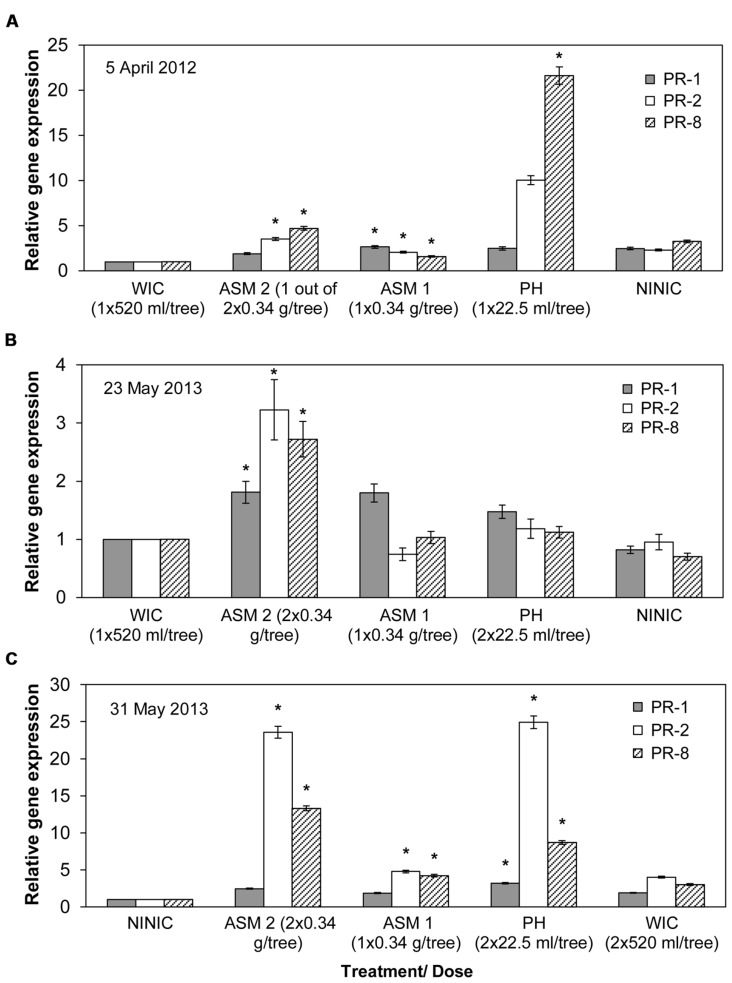
**Relative expression of PR-1, PR-2, and PR-8 genes in ‘Gala’ apple leaves tested by qRT-PCR**. Samples were collected in 2012 (A) and 2013 (B,C) following trunk injection/s of different compounds. PR, pathogenesis related genes; ASM, acibenzolar-*S*-methyl; PH, potassium salts of phosphorous acid (potassium phosphites); WIC, water injected control; NINIC, non-injected non-inoculated control. Mean gene expression followed by an asterisk (*) is significant relative to WIC or NINIC and normalized to actin gene (Pair Wise Fixed Reallocation Randomization test, α = 0.05). PR gene expression in **(C)** is shown in relation to NINIC since it was not significant in relation to WIC. Error bars represent SEM.

Compared to the water injected control, ASM injection into apple trees resulted in significant induction of PR-1, 2, and 8 genes in two consecutive years (**Figure 3**; Supplemental Table S3). On the other hand, PH injection significantly induced all three PR genes in 2013, and PR-8 gene in 2012 (**Figure 3**). SAR induction effect by ASM occurred 10 days after the first injection (DAFI) in 2012 (**Figure 3A**), and 22 DAFI and 1 day after the second injection (DASI) in 2013 (**Figure 3B**). SAR induction by PH in 2013 occurred 30 DAFI and 9 DASI (**Figure 3C**).

Various levels of induction of PR genes have been observed when injected with ASM and PH, compared to water injection control. In apple leaves collected on April 5, 2012, ASM injection induced the expression of all three PR genes from three- to almost fivefold higher than the water injected control, while PH induced PR-8 gene almost 22-fold (**Figure 3A**). In apple leaves collected on May 23, 2013, ASM 2 injection induced the expression of all three PR genes two- to almost threefold higher than the expression in water injected control (**Figure 3B**). In apple leaves collected on May 31, 2013, injection of ASM 1 induced the expression of PR-2 and 8 genes up to almost fivefold higher than the water injected control, while ASM 2 injection induced the expression of PR-2 and PR-8 genes to 13 and almost 24-fold higher than the non-injected non-inoculated control (**Figure 3C**). In addition, PH injection induced the expression of PR-1, 2, and 8 genes to about 3, 25, and 8-fold higher than the non-injected non-inoculated control (**Figure 3C**). PR gene expression on May 31, 2013 in relation to water injected control was not significant, showing the value of non-injected non-inoculated control in demonstrating PR gene expression after ASM and PH injections. This indicates that at this time point fire blight infection induced certain level of PR gene expression on control trees injected by water, which was enough to conceal the PR gene expression induced by ASM and PH injections. At all other time points in 2012 and 2013, significant induction of PR protein gene expression was rare (July 2, 2012) and mostly inconsistent among the tree replicates.

#### Gene expression in apple flowers

Gene expression on April 16, 2012, or 21 DAFI, showed that the injection of PH and ASM 2 only induced the expression of the PR-8 gene in vegetative flower parts (Supplemental Table S3). PH provided twofold and ASM provided fivefold induction of PR-8 expression in water injected control. No significant induction of the expression of all three PR genes was observed in flowers sampled on May 14, 2013.

## DISCUSSION

This study contributes new knowledge on fire blight management using tree injection as an alternative delivery approach for plant protective compounds. At short time periods between injection and inoculation, trunk-injected bactericides, and plant resistance inducers showed good potential in fire blight control on apple flowers and shoots. Since the injected compounds are affected by the tree physiology and interactions with tissues and sap, earlier injection dates allowing more time for compound translocation should improve their accumulation and efficiency in blossom blight control, both of which are hampered by small tissue volume and lower transpiration footprint of flowers. Higher dose of injected compound might be needed for long-lasting shoot blight control due to rapid increase in shoot tissue volume and higher transpiration footprint. This is the first study demonstrating significant fire blight suppression through resistance activation (SAR) indicated by induction of PR gene expression with injected ASM and PH on mature apple trees, under field conditions.

Control of blossom blight under medium and high disease pressures was consistently best with injected SS and PH, followed by ASM. However, injected SS was not as effective as its spray applications, which control blossom blight to incidences of only 0.2–3.5% (Sundin et al., 2009; McGhee and Sundin, 2011). This suggests that injected antibiotics either do not reduce bacterial populations on flowers as after topical application, because they do not reach the surface of stigmas, favorable for *E. amylovora* growth, or they do reach these surfaces but too late for better effect. However, once *E. amylovora* invaded the inner flower tissues, the injected bactericides most likely accumulated in the tissues and halted further advancement thus stopping the pathogen spread on other flowers and into the spurs and twigs.

Blossom blight control with injected PH and ASM occurred probably due to SAR which was expressed in nearby leaves before or after full bloom, depending on the year. However, PR genes in flowers *per se* were not expressed as consistently as in leaves and in both trial years. This can be explained by the fact that in *Malus* spp. vegetative flowers parts and later fruits have 10- to 100-fold lower frequency of stomata on epidermis in comparison to abaxial epidermis of leaves (Blanke and Lenz, 1989). Therefore, the transpiration rate per surface unit is much weaker, leading to slower accumulation of injected compounds in flowers than in leaves and hence delayed SAR expression which reduced blossom blight control potential. This delayed SAR expression in flowers was partially detected in 2012 and not at all in 2013, because bloom lasted longer in 2012 and because flowers were collected only at full bloom in both years. Previous research on ‘Golden Delicious’ showed that 2–4 ASM sprays (100 and 200 mg/L; 75, 150 and 200 mg of a.i./L) provided 3–52% of blossom blight control, while on ‘Rome Beauty’ 74–91% of control (Thomson et al., 1998; Brisset et al., 2000). Sprayed ASM (0.024 and 0.012%) on ‘James Grieve’ gave 56–68% of blossom blight control (Zeller and Zeller, 1998). Our 1–2 times injected ASM gave control of around 19–42%, indicating that injection does not improve the effect of this compound on flowers. The only investigation of blossom blight control with trunk-injection of plant resistance inducer, evaluated PCA, the free acid of prohexadione-calcium (Apogee, BASF Corp., Research Triangle Park, NC, USA; Düker and Kubiak, 2011). Injected PCA (10–40 mg/tree) provided 13.6–17.5% of blossom blight control on ‘White Transparent’ and ‘Gala Must’ apple trees (Düker and Kubiak, 2011). PCA also caused expected shoot stunting. All the above implies that injected compounds for blossom blight control must translocate and accumulate more rapidly in flowers to be effective, or should be injected much earlier allowing ample time for better compound accumulation and thus stronger effect on the pathogen. Optimizing the time and schedule of trunk injection(s) is an important consideration for maximizing the effect of compounds investigated in pest control on agricultural tree crops (Byrne et al., 2014).

Shoot blight incidence was best controlled with injected SS and then with ASM and PH, both of which induced resistance in leaves. Consistently, at the first date of disease rating, SS and PH in 2012 and all the injected compounds in 2013 showed better fire blight suppression on shoots than on flowers. The driver of this effect was probably the high transpiration rate of shoots which hold the largest leaf area in the apple canopy. This implies that shoots rapidly accumulate high amounts of injected compounds, controlling the disease early after injection. Weakening of control effects at later ratings can be explained by the compound dilution effect facilitated by tissue mass increase through shoot growth (Long et al., 1989), or metabolic processes leading to a.i. concentration decline (Lindquist, 1965). Further, unfavorable chemical properties of a.i.-s and their formulations, such as low water solubility and high organic carbon–water partitioning coefficient (Koc, ml/g; μg/g), which expresses the level of adhesion of a.i. to the carbon rich compounds in certain environment, could have hampered their translocation and accumulation. Koc is assumed as the key property determining compound mobility in xylem and the primary mechanism behind the reservoir effect in trunk after injection (Doccola et al., 2012; Aćimović et al., 2014b). Injected Arborfos (Mauget Inc., Arcadia, CA, USA), a PH generic, showed shoot blight control of 67% on inoculated ‘Paulared’ apple trees (Spitko, 2008). In our study, with the same dose per tree delivered in two split injections of PH, we achieved disease control of 23–65%. This implies that temporal dose splitting led to a weakening of shoot blight control by PH and most likely by ASM. On inoculated shoots, 3–6 ASM sprays (0.15 g/L) provided shoot blight control of 2.8 and 50.7%, respectively, while SS gave 56% control (Maxson and Jones, 1999). On naturally infected ‘Jonathan’ apple trees, six ASM sprays (75 mg/L) provided 50% of shoot blight control which was similar to SS with 57% of control (Maxson-Stein et al., 2002). We show that 1–2 injections of ASM provided only up to 27–30.9% of shoot blight control. Therefore, it seems that two-time injection does not significantly improve shoot blight control by ASM.

Excellent control of shoot blight severity by injected OTC implies that this compound in injectable formulation most likely limits systemic spread of *E. amylovora* in apple shoots. This is supported by the fact that in 2013, unlike in 2012, time between injection and inoculation was insufficient for OTC to achieve a spatially uniform distribution in the crown (Aćimović et al., 2014b), reach all the inoculated shoots, and impact the pathogen. Therefore, infections have progressed to about 20% on June 10. However, after that, OTC stopped the infections and this effect lasted until the end of the experiment. Even though OTC is bacteriostatic, we show that this antibiotic injected once per season has the ability to express its effectiveness longer and better than after spraying (Johnson and Stockwell, 1998; McManus et al., 2002). Hence, trunk injection enhanced the activity of OTC in shoot blight control.

Finally, we show that on mature apple trees the injected PH can induce SAR in apple leaves and confirm this effect for ASM. SAR allowing or aiding disease control was implicated after evaluation of PH on different plant species (Smillie et al., 1989). PH did not show SAR induction in 2012, probably because the leaf sampling times were too far apart or too close to the injection dates, thus not allowing the detection of induced PR gene expression. Hence, at sampling times in 2012, accumulated PH doses in leaves were most likely either too low to cause significant induction of PR gene expression or the induction of gene expression had already ceased.

Significant PR gene expression, indicating on induction of SAR, occurred before the detected fire blight control effects, which were more persistent in flowers than in shoots, and showed that trunk injections were properly timed for disease control. The time separating SAR induction and fire blight control effects indicate that probably the myriad of synthesized and accumulated defensive compounds suppressed the disease. Injected ASM consistently induced the expression of PR-1, PR-2, and PR-8 protein genes in both years of the study. Based on research on tobacco including PR-1, then on apple including PR-2, and on cucumber including PR-8, these genes code for proteins with an anti-oomycete-activity, β-1,3-glucanase with hydrolase activity (on cell walls of fungi and oomycetes), and class III chitinase, i.e., lysozyme with hydrolysis activity (on cell walls of bacteria), respectively (Maxson-Stein et al., 2002). The same three PR genes were significantly expressed in ‘Jonathan’ apple seedlings after ASM spray treatment (250 mg a.i./L; Maxson-Stein et al., 2002). Similar results were also achieved with ASM (100 and 200 mg a.i./L) on ‘Golden Delicious’ apple seedlings (Brisset et al., 2000). However, in 1 year-old ‘Gala’ apple trees treated with ASM (250 mg a.i./L), no significant induction in expression of PR-1a, PR-2, and PR-8 genes was detected in shoots (Bonasera et al., 2006). In this study, PR-2 and PR-8 were induced only in shoots after *E. amylovora* inoculation, and the difference from results of induction in seedlings was attributed to the differences in plant development stage and responses of the treated tissues. In the present study, we show that very soon, i.e., 1–10 days after injection, ASM induces PR gene expression in leaves on mature ‘Gala’ apple trees.

In summary, our fire blight control experiments indicated that injected compounds accumulated at sufficient amounts in the apple tree canopy and expressed their putative effects on the plant or the pathogen. The results imply that accumulation of injected compound in the crown is a time dependent process, potentially not fully exposing epiphytic bacterial populations to the compound and requiring much earlier times of injection for better accumulation and effect of the compound on the pathogen. Injected SS provided the best fire blight control but, excluding OTC, ASM, and SS, were probably hampered in translocation, accumulation, and thus activity, by formulations for topical application. Injected ASM provided consistent SAR induction but weaker fire blight control in mature apple trees, while PH, besides good control, showed that these compounds induce SAR. Overall, the results indicate that tree injection could decrease antibiotic usage in the open environment, thus reducing the potential for side effects to the environment.

## Conflict of Interest Statement

The authors declare that the research was conducted in the absence of any commercial or financial relationships that could be construed as a potential conflict of interest.
