# Supplementary material for: Control of fire blight (Erwinia amylovora) on apple trees with trunk-injected plant resistance inducers and antibiotics and assessment of induction of pathogenesis-related protein genes
Source: Front Plant Sci. 2015 Feb 10;6:16. doi: 10.3389/fpls.2015.00016 (PMC4323746; doi:10.3389/fpls.2015.00016)
Supplement: Supplementary file 1 [file Supplemental_Tables.PDF]

# *Supplementary Material*

# Control of fire blight (*Erwinia amylovora*) on apple trees with trunk-injected plant resistance inducers and antibiotics and assessment of induction of pathogenesis-related protein genes

**Srđan G. Aćimović[1,2*], Quan Zeng[1], Gayle C. McGhee[1], George W. Sundin[1] and John C. Wise[2]**

[1]Tree Fruit Pathology Laboratory, Department of Plant, Soil and Microbial Sciences, Michigan State University, East Lansing, MI, USA.
[2]Applied Insecticide Toxicology Laboratory, Department of Entomology, Michigan State University, East Lansing, MI, USA.

***Correspondence:** Srđan G. Aćimović, Michigan State University, Department of Plant, Soil and Microbial Sciences, 206A Center for Integrated Plant Systems, 578 Wilson Road, East Lansing, MI 48824-1311, USA, e-mail: acimovic@msu.edu

## 1. Supplementary Tables

Supplemental Table S1. Sequences of primers used for PCR amplification of genes in cDNA (Maxson-Stein et al., 2002).

| Target gene | Primer ID | Primer sequence |
|---|---|---|
| PR-1 | aj708 | GTAGGCGTTGGTCCCTTGAC |
| PR-1 | aj709 | GATTGCAGTCGCCAACATGT |
| PR-2 | aj778 | TCCGATGCCATTGCTTTTG |
| PR-2 | aj779 | TTATGGACGAAACGGCAACA |
| PR-8 | aj780 | CTCTTTTGAGCAGTTGGAACCA |
| PR-8 | aj781 | TGCCGGTAACCCCATGAA |
| Actin | aj748 | AACTTCGTGTTGCTCCTGAAGAG |
| Actin | aj749 | CAGTAGTACGACCACTGGCATAGAG |

*Copyright © 2015 Aćimović, Zeng, McGhee, Sundin and Wise*

Supplemental Table S2. The main effects and their interactions after trunk-injection of apple trees with compounds for control of fire blight in 2012 and 2013.

| Fire blight stage | Year/Date | Main effects | *F* | DF | *p* value |
|---|---|---|---|---|---|
| **Blossom blight incidence** | **2012** | Treatment | 3.13 | 15 | **≤0.0393** |
| | | Time | 5.76 | 23.5 | **≤0.0092** |
| | | Treatment*Time | 0.49 | 26.7 | ≥0.8817 |
| | **2013** | Treatment | 28.76 | 15 | **≤0.0001** |
| | | Time | 14.04 | 30 | **≤0.0001** |
| | | Treatment*Time | 0.65 | 30 | ≥0.7315 |
| **Shoot blight incidence** | **29 May 2012** | Treatment | 6.74 | 10.1 | **≤0.0052** |
| | **2013** | Treatment | 10.10 | 17.5 | **≤0.0002** |
| | | Time | 46.54 | 31.4 | **≤0.0001** |
| | | Treatment*Time | 1.71 | 31.5 | ≥0.1344 |
| **Shoot blight severity** | **2012** | Treatment | 10.68 | 6.1 | **≤0.0167** |
| | | Time | 4.47 | 4.56 | ≥0.0744 |
| | | Treatment*Time | 1.68 | 4.56 | ≥0.2981 |
| | **2013** | Treatment | 49.14 | 6.8 | **≤0.0002** |
| | | Time | 12.5 | 29.8 | **≤0.0001** |
| | | Treatment*Time | 9.09 | 29.8 | **≤0.0001** |

Copyright © 2015 Aćimović, Zeng, McGhee, Sundin and Wise 

Supplemental Table S3. Significance in the expression of pathogenesis related (PR) genes in apple leaves and flowers after trunk-injection of 'Gala' trees with plant resistance inducers for control of fire blight. Significance was relative to water injected control or to non-injected non-inoculated control and normalized to actin gene (Pair Wise Fixed Reallocation Randomization test, α=0.05). ASM, acibenzolar-S-methyl; PH, potassium salts of phosphorous acid.

| Plant organ | Date | Compound | Protein gene | *p* value |
|---|---|---|---|---|
| Leaves | 5 April 2012 | ASM | PR-1 | **≤0.042** |
| | | | PR-2 | **≤0.042** |
| | | | PR-8 | **≤0.042** |
| | | PH | PR-1 | ≥0.946 |
| | | | PR-2 | ≥0.946 |
| | | | PR-8 | **≤0.001** |
| | 23 May 2013 | ASM | PR-1 | **≤0.046** |
| | | | PR-2 | **≤0.046** |
| | | | PR-8 | **≤0.046** |
| | 31 May 2013 | ASM | PR-1 | ≥0.492 |
| | | | PR-2 | **≤0.042** |
| | | | PR-8 | **≤0.042** |
| | | PH | PR-1 | **≤0.045** |
| | | | PR-2 | **≤0.045** |
| | | | PR-8 | **≤0.045** |
| Flowers | 16 April 2012 | ASM | PR-1 | ≥0.431 |
| | | | PR-2 | ≥0.894 |
| | | | PR-8 | **≤0.042** |
| | | PH | PR-1 | ≥0.603 |
| | | | PR-2 | ≥0.946 |
| | | | PR-8 | **≤0.001** |

## 2. References

Maxson-Stein, K., He, S. Y., Hammerschmidt, R., and Jones, A. L. (2002). Effect of treating apple trees with acibenzolar-S-methyl on fire blight and expression of pathogenesis-related protein genes. *Plant Dis.* 86, 785–790. doi:10.1094/PDIS.2002.86.7.785.

*Copyright © 2015 Aćimović, Zeng, McGhee, Sundin and Wise*
